# Eribulin-based neoadjuvant chemotherapy for triple-negative breast cancer patients stratified by homologous recombination deficiency status: a multicenter randomized phase II clinical trial

**DOI:** 10.1007/s10549-021-06184-w

**Published:** 2021-03-25

**Authors:** Norikazu Masuda, Hiroko Bando, Takashi Yamanaka, Takayuki Kadoya, Masato Takahashi, Shigenori E. Nagai, Shoichiro Ohtani, Tomoyuki Aruga, Eiji Suzuki, Yuichiro Kikawa, Hiroyuki Yasojima, Hiroi Kasai, Hiroshi Ishiguro, Hidetaka Kawabata, Satoshi Morita, Hironori Haga, Tatsuki R. Kataoka, Ryuji Uozumi, Shinji Ohno, Masakazu Toi

**Affiliations:** 1 Department of Surgery, Breast Oncology, NHO Osaka National Hospital, 2-1-14 Hoenzaka, Chuou-ku, Osaka, Japan; 2 Breast and Endocrine Surgery, Faculty of Medicine, University of Tsukuba, Ibaraki, Japan; 3 Breast and Endocrine Surgery, Kanagawa Cancer Center, Kanagawa, Japan; 4 Department of Breast Surgery, Hiroshima University Hospital, Hiroshima University, Hiroshima, Japan; 5 Department of Breast Surgery, NHO Hokkaido Cancer Center, Hokkaido, Japan; 6 Division of Breast Oncology, Saitama Cancer Center, Saitama, Japan; 7 Department of Breast Surgery, Hiroshima City Hiroshima Citizens Hospital, Hiroshima, Japan; 8 Breast Surgery Division, Tokyo Metropolitan Cancer and Infectious Diseases Center Komagome Hospital, Tokyo, Japan; 9 Breast Cancer Unit, Kyoto University Hospital, Graduate School of Medicine, Kyoto University, Kyoto, Japan; 10 Department of Breast Surgery, Kobe City Medical Center General Hospital, Hyogo, Japan; 11 Institute for Advancement of Clinical and Translational Science, Kyoto University Hospital, Kyoto, Japan; 12 Breast Oncology Service, Saitama Medical University International Medical Center, Saitama, Japan; 13 Department of Breast and Endocrine Surgery, Toranomon Hospital, Tokyo, Japan; 14 Department of Biomedical Statistics and Bioinformatics, Kyoto University Graduate School of Medicine, Kyoto, Japan; 15 Department of Diagnostic Pathology, Kyoto University Hospital, Kyoto, Japan; 16 Breast Oncology Center, The Cancer Institute Hospital of JFCR, Tokyo, Japan

**Keywords:** *BRCA* mutation status, Eribulin, Homologous recombination deficiency score, Neoadjuvant chemotherapy, Pathological complete response, Triple-negative breast cancer

## Abstract

**Purpose:**

To investigate clinical usefulness of eribulin-based neoadjuvant chemotherapy in triple-negative breast cancer (TNBC) patients.

**Methods:**

Patients in group A (aged < 65 years with homologous recombination deficiency, HRD, score ≥ 42, or those at any age with germline *BRCA* mutation, gBRCAm) were randomized to 4 cycles of paclitaxel plus carboplatin (group A1) or eribulin plus carboplatin (group A2), followed by 4 cycles of anthracycline. Patients in group B (aged < 65 years with HRD score < 42, or aged ≥ 65 years without gBRCAm) were randomized to 6 cycles of eribulin plus cyclophosphamide (group B1) or eribulin plus capecitabine (group B2); non-responders to the first 4 cycles of the eribulin-based therapy received anthracycline. Primary endpoint was pCR rate (ypT0-is, ypN0; centrally confirmed). Main secondary endpoint was safety.

**Results:**

The full analysis set comprised 99 patients. The pCR rate was 65% (90% CI, 46%–81%) and 45% (27%–65%) in groups A1 and A2, respectively, and 19% (8%–35%) in both groups B1 and B2. No major difference was seen in secondary endpoints, but peripheral neuropathy incidence was 74% in group A1, whereas it was 32%, 22%, and 26% in groups A2, B1, and B2, respectively.

**Conclusions:**

In patients aged < 65 years with high HRD score or gBRCAm, weekly paclitaxel plus carboplatin and eribulin plus carboplatin followed by anthracycline resulted in a pCR rate of > 60% and > 40%, respectively, suggesting potential usefulness of patient stratification using HRD; pCR tended to be low in patients with HRD-negative tumors. Neurotoxicity was less frequent with the eribulin-based regimen. *Trial registration*:The study has been registered with the University Hospital Medical Information Network Clinical Trials Registry (http://www.umin.ac.jp/ctr/index-j.htm) with unique trial number UMIN000023162. The Japan Breast Cancer Research Group trial number is JBCRG-22.

**Supplementary Information:**

The online version contains supplementary material available at 10.1007/s10549-021-06184-w.

## Introduction

Triple-negative breast cancer (TNBC), which accounts for about 10–20% of breast cancers, is an aggressive subtype with a poor prognosis [1, 2]. Because it lacks specific targets for treatment [1, 2], research is being directed toward improving neoadjuvant chemotherapy for TNBC. Pathological complete response (pCR) correlates with favorable prognosis [3–6] and is used to gauge the success of new regimens. Patients with TNBC typically receive anthracycline- and taxane-based perioperative regimens. However, these have limited success [7], so new regimens including addition of chemotherapeutic agents to existing regimens have been proposed as a way to improve pCR rate [8, 9].

Previously, our research group investigated neoadjuvant chemotherapy regimens with an anthracycline–docetaxel combination [10–13]. For TNBC, we conducted a phase II study of neoadjuvant metronomic chemotherapy with paclitaxel, cyclophosphamide, and capecitabine followed by an anthracycline-containing regimen (JBCRG-13), which resulted in a high pCR rate (intent-to-treat population, 47.5%) with acceptable tolerability [14].

As our next research target, we consider treatment with eribulin, a novel microtubule inhibitor, to be potentially useful as an alternative to taxane-based regimens for TNBC. Eribulin disrupts mitosis by inhibiting the microtubule growth phase without affecting the shortening phase [15–18]. Distinct effects of eribulin include vascular remodeling [19, 20] and suppression of epithelial–mesenchymal transition [21, 22]. These unique mechanisms of action make it efficacious in cases of residual cancer or resistance to standard neoadjuvant chemotherapy including taxanes [19, 23, 24], and it particularly benefits TNBC patients [25]. Moreover, the incidence of peripheral neuropathy, a frequent adverse effect of taxanes, tends to be low with eribulin [22, 24, 25].

One of the aims of the present study was to use response predictors to identify patients most likely to benefit from chemotherapy. Therefore, we stratified TNBC patients by homologous recombination deficiency (HRD) score [26] and germline *BRCA* mutation (gBRCAm) status [27], both of which are effective predictors for pCR, particularly when patients are treated with platinum-containing chemotherapy. HRD scores correlate strongly with *BRCA1/2* deficiency, regardless of breast cancer subtype [28]. We evaluated their usefulness as response predictors in TNBC patients receiving carboplatin in combination with standard paclitaxel- or eribulin-based regimens, for the following reasons. Addition of carboplatin to standard neoadjuvant anthracycline- and taxane-based chemotherapy has been shown to improve pCR rate in patients with HER2-negative breast cancer [29] or TNBC [8, 30, 31]. Alternatively, eribulin plus carboplatin could be used as neoadjuvant chemotherapy for TNBC, to avoid peripheral neuropathy caused by taxanes. In a study in which patients with early-stage TNBC received four cycles of carboplatin plus eribulin, pCR was confirmed in 13 (43.3%) of 30 patients; 12 of 26 patients had HRD-positive tumors, and of them, 9 patients achieved pCR, suggesting that HRD-positive status may predict pCR in TNBC patients receiving this treatment [32].

Additionally, for elderly patients or those without gBRCAm, we aimed to investigate a potentially less toxic regimen of eribulin combined with capecitabine or cyclophosphamide. To avoid cardiotoxicity caused by anthracyclines [33, 34], non-anthracycline regimens are being investigated. One example is docetaxel plus cyclophosphamide (TC) [35–37]; however, TC is insufficiently efficacious against certain cancer subtypes (e.g., basal-like/BRCAness phenotype) [36]. Therefore, in elderly patients and those without gBRCAm, we investigated treatment de-escalation by using eribulin in combination with cyclophosphamide or capecitabine. The safety of eribulin plus capecitabine had previously been confirmed in a phase I study (JBCRG-18) [38].

Our primary aim of the present study was to investigate the clinical usefulness of eribulin-containing neoadjuvant regimens for TNBC by means of stratification by HRD score and gBRCAm status. We also aimed to explore the option of potentially less toxic regimens for elderly TNBC patients.

## Methods

### Study design

**Fig. 1:**
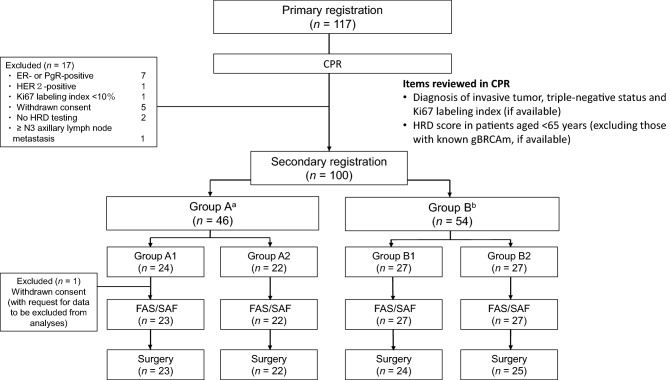
Study flow and patient disposition. ^a^Group A included patients aged < 65 years and with HRD score ≥ 42, and all patients with gBRCAm. ^b^Group B included patients aged < 65 years and with HRD score < 42, and all patients aged ≥ 65 years except those with gBRCAm. gBRCAm, germline BRCA mutation; CPR, centralized pathologic review; ER, estrogen receptor; FAS, full analysis set; HER2, human epidermal growth factor receptor 2; HRD, homologous recombination deficiency; PgR, progesterone receptor; SAF, safety analysis set

In this multicenter randomized parallel-group phase II clinical trial, the main inclusion criteria were: diagnosis of primary TNBC (resectable tumor; cT1c–cT3, cN0–cN1, and cM0) confirmed as invasive by centralized pathologic review (CPR); diameter ≤ 70 mm; Ki67 labeling index ≥ 10% (if available). Patients who met the criteria for primary registration were subjected to CPR (based at Kyoto University Hospital, Kyoto, Japan), and those who met the additional criteria for secondary registration were enrolled (Fig. 1). Details of inclusion/exclusion criteria are provided as Supplementary information.

### Randomization and allocation to treatment groups

Random allocation was performed by the registration center, using the minimization method. The adjustment factors were age at secondary registration (< 50 years or ≥ 50 years), tumor diameter (T; < 30 mm or ≥ 30 mm), axillary lymph node (ALN) status (N; cN0, or cN( +)), and Ki67 labeling index (< 30% or ≥ 30%).

Patients were allocated to two groups based on HRD score, gBRCAm status (if available), and age at time of informed consent. Group A included patients aged < 65 years and with HRD score ≥ 42, and all patients with gBRCAm. Group B included patients aged < 65 years and with HRD score < 42, and all patients aged ≥ 65 years except those with gBRCAm. The rationale for patient allocation to groups A and B was based on the expectation of a good response to platinum-containing chemotherapy in patients with high HRD score or gBRCAm (group A) [27, 32, 39, 40] and the investigation of potentially less toxic regimens for older patients (group B).

### Treatment

**Fig. 2:**
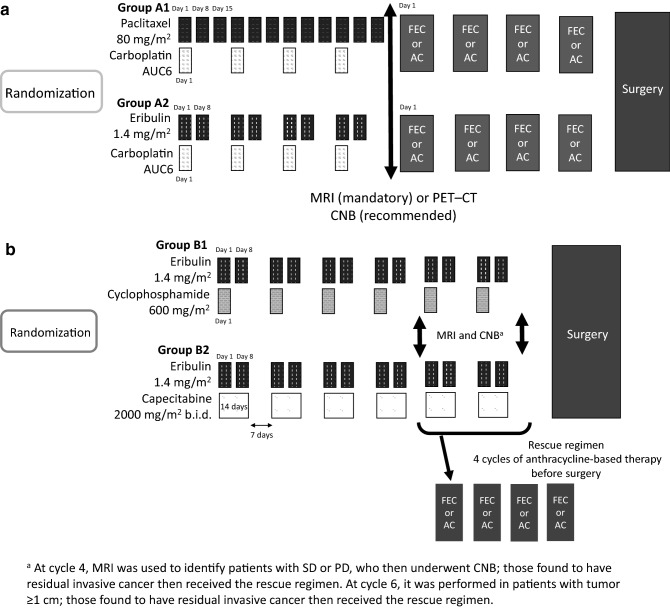
Dosing schedule. **a** Patients in group A1 received 4 cycles of combination therapy with paclitaxel (80 mg/m^2^) on days 1, 8, and 15 of each cycle and carboplatin (AUC6) on day 1. Patients in group A2 received 4 cycles of combination therapy with eribulin (1.4 mg/m^2^) on days 1 and 8 of each cycle and carboplatin (AUC6) on day 1. Depending on the antitumor effects of the initial therapy, evaluated at cycle 4, patients subsequently received the anthracycline-based regimen before surgery, or discontinued chemotherapy and underwent surgery. The anthracycline-based regimen comprised FEC, consisting of 5-fluorouracil (500 mg/m^2^), epirubicin (100 mg/m^2^), and cyclophosphamide (500 mg/m^2^); or AC, consisting of doxorubicin (60 mg/m^2^) and cyclophosphamide (600 mg/m^2^), on day 1 of each cycle. **b** Patients in group B1 received 4 cycles of eribulin (1.4 mg/m^2^) on days 1 and 8 of each cycle and cyclophosphamide (600 mg/m^2^) on day 1. Patients in group B2 received 4 cycles of eribulin (1.4 mg/m^2^) on days 1 and 8 of each cycle and capecitabine (2000 mg/m^2^/day) administered orally twice daily on days 1–14. Depending on the antitumor effects, assessed at cycle 4, patients in groups B1 and B2 received an additional 2 cycles of the eribulin-based regimen before surgery, or underwent surgery after receiving the rescue regimen (FEC or AC as described for group A). AC, doxorubicin–cyclophosphamide regimen; CNB, core needle biopsy; CT, computed tomography; FEC, 5-fluorouracil–epirubicin–cyclophosphamide regimen; MRI, magnetic resonance imaging; PD, progressive disease; PET, positron emission tomography; SD, stable disease

Figure 2 shows the dosing schedule. In group A, patients were randomized to either group A1, receiving a standard taxane and platinum regimen (paclitaxel plus carboplatin) followed by an anthracycline-based regimen (5-fluorouracil–epirubicin–cyclophosphamide, FEC or doxorubicin–cyclophosphamide, AC), or group A2, receiving the same regimen except with eribulin instead of paclitaxel. The antitumor effects of the initial therapy were evaluated at cycle 4 by MRI (mandatory) or PET–CT. Core needle biopsy (CNB) was also performed at cycle 4, when possible, to determine pathological response.

In group B, patients were randomized to either group B1, receiving a regimen combining eribulin with cyclophosphamide, or group B2, receiving a regimen combining eribulin with capecitabine. At cycle 4, the antitumor effects of the initial therapy were evaluated by MRI or PET–CT to determine complete response (CR), partial response (PR) stable disease (SD), or progressive disease (PD) and determine tumor diameter (< 10 mm or ≥ 10 mm). In cases of SD or PD, CNB was performed to determine whether patients had residual invasive tumor. Patients with CR or PR, and those with SD but no residual invasive tumor, received two further cycles of the eribulin-based regimen before surgery. Patients with SD and confirmation of residual invasive tumor were switched to 4 cycles of FEC or AC (rescue regimen) before surgery. Patients with PD were switched to the rescue regimen followed by surgery, or surgery. For the patients who received the additional cycles of eribulin plus cyclophosphamide or capecitabine, the antitumor effects were evaluated at cycle 6. Patients with tumor diameter < 10 mm, or with tumor diameter ≥ 10 mm and no residual invasive tumor, underwent surgery. Patients with tumor diameter ≥ 10 mm and confirmation of residual invasive tumor received the rescue regimen followed by surgery, or surgery.

The treatment was initiated within 2 weeks of secondary registration. Each cycle lasted 21 days.

### Surgery

Mastectomy or breast-conserving surgery (BCS) was carried out as a curative surgery within 11 weeks of the start of the final cycle of chemotherapy. The decision of which surgical procedure to choose was based on the spread of residual tumor by imaging and the preference of the individual patient, with the aim of obtaining adequate negative margins.

### Assessment of efficacy and safety

Pathologic response was assessed by examination (at each participating institution and by the CPR committee) of hematoxylin–eosin-stained tissue samples removed during surgery (operative specimens). Based on examination of the primary lesion, pathologic response was classified as strict pCR (SpCR; no residual tumor), comprehensive pCR (CpCR; SpCR or residual ductal carcinoma in situ), or quasi pCR (QpCR; CpCR plus near pCR [small foci of cancer cells]). Specimens from ALNs were also examined, and metastasis in the ALNs (including isolated tumor cell clusters) of ≤ 0.2 mm was defined as ypN0. Clinical response was assessed based on the Response Evaluation Criteria in Solid Tumors version 1.1.

Adverse events (AEs), defined as any unfavorable or unintended sign that may or may not be associated with the study treatment, were recorded and graded according to the National Cancer Institute Common Terminology Criteria for Adverse Events, version 4.0 (Japanese Clinical Oncology Group edition) [41].

### Endpoints

The primary endpoint was CpCR (ypT0ypN0 or ypTisypN0) rate. Secondary endpoints for efficacy included proportion of patients with SpCR or SpCR plus ypN0, CpCR, and QpCR or QpCR plus ypN0; clinical response rate (i.e., response rate to the eribulin-based regimen and the anthracycline-based regimen, and overall response rate); BCS rate (BCSR); and proportion of patients whose ALN status had become negative by the time of surgery. Tumor regression from baseline (or the start of FEC or AC) to completion of neoadjuvant chemotherapy was assessed. Secondary endpoints for safety were incidence of AEs, treatment completion rate, and relative dose intensity.

### Statistical analyses

When setting an α error of 0.05 (one-sided) and a power of 80% with a threshold pCR rate of 0.40 and an expected pCR rate of 0.60 for group A, and a threshold pCR rate of 0.35 and an expected pCR rate of 0.55 for group B, the maximum required numbers of patients were calculated as 88 for group A (44 for each of groups A1/A2) and 90 for group B (45 for each of groups B1/B2). Allowing for patients excluded from analyses, the target number was determined as 100 for each of groups A and B.

Efficacy was evaluated using data from all patients in the full analysis set (FAS), defined as all patients who fulfilled the inclusion criteria and received ≥ 1 dose of a study drug. Safety was evaluated using data from the safety analysis set (SAF), comprising all patients who received ≥ 1 dose of a study drug.

For the primary endpoint (i.e., pCR rate), and the secondary endpoints of SpCR rate and SpCR plus ypN0 rate, overall response rate, and BCSR, point estimates and two-sided 90% or 95% Clopper–Pearson exact CIs were calculated for groups A1, A2, B1, and B2. Because our primary aim in this phase II trial was to assess the therapeutic efficacy of candidate chemotherapeutic regimens for TNBC, our focus was the efficacy endpoints for each group rather than intergroup comparisons. However, for exploratory purposes, we also calculated *P* values using Fisher’s exact test for comparisons within groups A and B (i.e., group A1 versus A2, and group B1 versus B2).

Statistical analyses were performed using SAS software, version 9.4 (SAS Institute, Cary, NC, USA).

## Results

### Participant flow

Patients were recruited in Japan between February 2017 and January 2019. Recruitment ended when 100 patients had been found to have met the criteria for secondary registration.

Of the 117 patients initially recruited, 100 met the criteria for secondary registration. One patient in group A1 withdrew consent after secondary registration. In accordance with the patient’s request, their data were excluded from all analyses. Consequently, both the FAS and SAF comprised 99 patients (Fig. 1).

### Baseline data

Most baseline characteristics were well balanced between the groups (Table 1). Age at secondary registration was higher in group B than in group A, due to age being a factor in the patient allocation to treatment groups. Group A included no patients aged ≥ 65 years. Histological grade tended to be higher in group A than in group B. All patients had invasive ductal carcinoma.

**Table 1:** Baseline patient characteristics^a^

Characteristic	Group A1 (*n* = 23)	Group A2 (*n* = 22)	Group B1 (*n* = 27)	Group B2 (*n* = 27)
**Age at secondary registration, years**				
Mean ± SD	47.4 ± 11.0	47.3 ± 11.3	59.3 ± 8.4	58.8 ± 9.1
Median (minimum, maximum)	44.0 (28, 64)	47.5 (26, 63)	59.0 (35, 70)	60.0 (37, 70)
**Age category at secondary registration, years**				
< 50	14 (61)	13 (59)	3 (11)	3 (11)
≥ 50	9 (39)	9 (41)	24 (89)	24 (89)
**TNM classification: T (primary lesion)**				
T1c	10 (43)	6 (27)	2 (7)	7 (26)
T2	12 (52)	14 (64)	23 (85)	17 (63)
T3	1 (4)	2 (9)	2 (7)	3 (11)
**Tumor size (primary lesion), mm**				
Mean ± SD	27.4 ± 12.2	31.0 ± 13.9	33.7 ± 13.8	32.1 ± 17.1
Median (minimum, maximum)	23.0 (13, 60)	26.5 (13, 64)	30.0 (12, 70)	26.0 (12, 92)
**TNM classification: N (regional lymph node)**				
N0	15 (65)	14 (64)	16 (59)	17 (63)
N1	8 (35)	8 (36)	11 (41)	10 (37)
**Type of invasive carcinoma**				
Ductal	23 (100)	22 (100)	27 (100)	27 (100)
Lobular	0	0	0	0
**Histological grade (B&R)**				
1	1 (4)	0	0	3 (11)
2	3 (13)	4 (18)	8 (30)	8 (30)
3	16 (70)	17 (77)	18 (67)	12 (44)
**HER2 status**^**b**^				
0	21 (91)	14 (64)	19 (70)	18 (67)
1 +	2 (9)	7 (32)	5 (19)	6 (22)
2 +	0	0	3 (11)	3 (11)
**Ki67 index, %**^**c**^				
*n*	23	22	27	27
Mean ± SD	55.1 ± 21.4	66.7 ± 16.9	55.3 ± 23.0	50.0 ± 19.9
Median (minimum, maximum)	58.0 (20.2, 92.4)	66.2 (36.4, 89.6)	51.6 (16.4, 90.4)	48.0 (16.2, 82.0)
**Ki67 index, category**^**b**^				
< 30%	3 (13)	0	5 (19)	6 (22)
≥ 30%	20 (87)	22 (100)	22 (81)	21 (78)

B&R, Bloom and Richardson grading system; HER2, human epidermal growth factor receptor 2

^a^Values are *n* (%) unless otherwise stated

^b^Confirmed immunohistochemically by centralized pathologic review

^c^Confirmed by centralized pathologic review

### Completion of planned treatment

#### Group A

Mean relative dose intensity was 87.1% for paclitaxel (group A1) and 83.9% for eribulin (group A2). For carboplatin, it was 96.4% and 96.6% in groups A1 and A2, respectively. After initially receiving ≤ 4 cycles of the paclitaxel- or eribulin-based regimen, 96% (22/23) and 95% (21/22) of patients in groups A1 and A2, respectively, received ≥ 1 cycle of one of the subsequent anthracycline-based regimens.

#### Group B

In groups B1 and B2, 48% (13/27) and 56% (15/27) of patients, respectively, completed all 6 cycles of the planned eribulin-based therapy. Details of the completion of planned treatment are summarized in Supplementary Fig. S1.

### Primary endpoint

Table 2 shows the proportion of patients achieving pCR. In group A1, 15 of 23 patients (65%; 90% CI, 46%–81%) achieved pCR. In group A2, 10 of 22 patients (45%; 90% CI, 27%–65%) achieved pCR; the pCR rate for group A1 met its primary objective (lower bound of 90% CI, > 40%). The pCR rate for groups B1 and B2 (19%, 90% CI, 8%–35% for each group) was below the threshold pCR rate (35%) predefined for group B.

**Table 2:** Pathological complete response (pCR; ypT0ypN0 or ypTisypN0) rate (primary endpoint): the proportion of patients achieving pCR

Group	No. of patients achieving pCR	pCR rate, % (90% CI^a^)	*P*^b^
A1 (*n* = 23)	15	65 (46–81)	0.24
A2 (*n* = 22)	10	45 (27–65)	
B1 (*n* = 27)	5	19 (8–35)	1.00
B2 (*n* = 27)	5	19 (8–35)	

^a^Clopper–Pearson exact confidence interval

^b^Fisher’s exact test: group A1 versus group A2 or group B1 versus group B2

### Secondary efficacy endpoints

#### Pathological response and clinical response

Table 3 shows the proportion of patients achieving SpCR plus pN0, CpCR, and QpCR plus pN0. Table 4 shows the results for clinical response at cycle 4 and completion of neoadjuvant chemotherapy.

**Table 3:** Strict pathological complete response (SpCR) plus pN0, comprehensive pathological complete response (CpCR), and quasi pathological complete response (QpCR) plus pN0 rates (secondary endpoints for efficacy)

Variable	Group A1 (*n* = 23)	Group A2 (*n* = 22)	*P*^b^	Group B1 (*n* = 27)	Group B2 (*n* = 27)	*P*^b^
No. of patients achieving SpCR plus pN0	14	8		2	5	
SpCR plus pN0 rate, % (90% CI^a^)	61 (42–78)	36 (20–56)	0.14	7 (1–22)	19 (8–35)	0.42
No. of patients achieving CpCR	16	12		6	7	
CpCR rate, % (90% CI^a^)	70 (50–85)	55 (35–73)	0.37	22 (10–39)	26 (13–43)	1.00
No. of patients achieving QpCR plus pN0	16	11		8	6	
QpCR plus pN0 rate, % (90% CI^a^)	70 (50–85)	50 (31–69)	0.23	30 (16–47)	22 (10–39)	0.76

^a^Clopper–Pearson exact confidence interval

^b^Fisher’s exact test

**Table 4:** Clinical response and overall response rate (confirmed by MRI or contrast CT examination)

Group	No. of patients achieving clinical response^c^					Overall response rate, % (95% CI^a^)	*P*^b^
	CR	PR	SD	PD	NE^d^		
**At cycle 4**							
A1 (*n* = 23)	8	13	1	0	1	91 (72–99)	0.41
A2 (*n* = 22)	6	12	2	1	1	82 (60–95)	
B1 (*n* = 27)	2	12	8	4	1	52 (32–71)	1.00
B2 (*n* = 27)	3	11	11	2	0	52 (32–71)	
**At completion of neoadjuvant chemotherapy**							
A1 (*n* = 23)	11	9	1	0	2	87 (66–97)	0.46
A2 (*n* = 22)	9	8	1	2	2	77 (55–92)	
B1 (*n* = 27)^e^	4	11	4	6	2	56 (35–75)	0.58
B2 (*n* = 27)^e^	5	13	5	3	1	67 (46–84)	

CR, complete response; NE, not evaluable; PD, progressive disease; PR, partial response; SD, stable disease

^a^Clopper–Pearson exact confidence interval

^b^Fisher’s exact test: group A1 versus A2 or group B1 versus group B2

^c^Patients who dropped out prior to cycle 4 or completion of neoadjuvant chemotherapy were considered not to have had a response (non-responder imputation)

^d^Data were not evaluable because treatment was switched to surgery; the target lesion lymph node was located outside the imaging area or not detected before the start of treatment; or the imaging test was not performed at the evaluation times

^e^In groups B1 and B2, 13 and 15 patients, respectively, completed the planned 6 cycles of treatment; 9 patients in each of groups B1 and B2 were switched to receive anthracycline-based regimens based on imaging test results at cycle 4

#### Tumor response

**Fig. 3:**
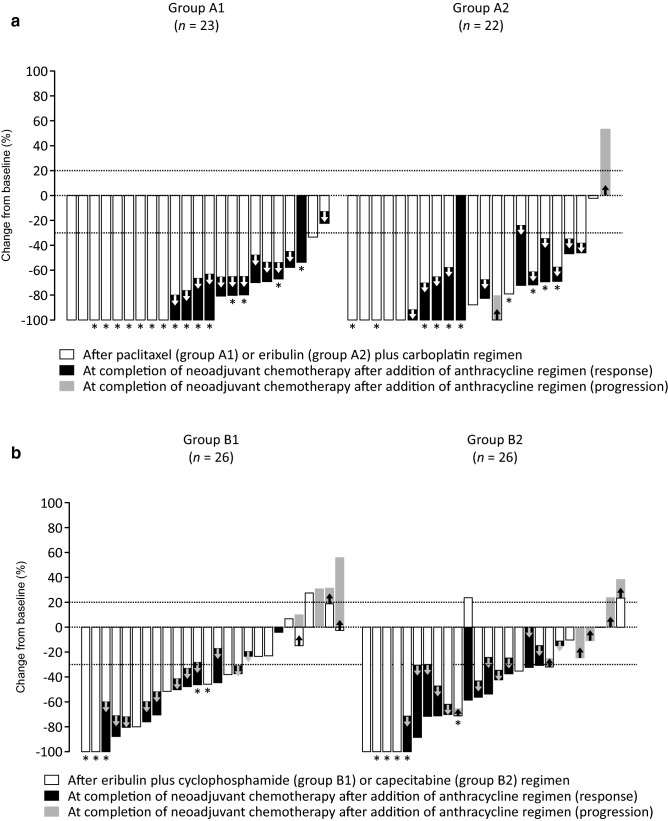
Waterfall plots showing change in tumor size from baseline **a** in group A1 (*n* = 23) and group A2 (*n* = 22) after the initial paclitaxel or eribulin plus carboplatin regimen and at completion of neoadjuvant chemotherapy, and **b** in group B1 (*n* = 26) and group B2 (*n* = 26) after the eribulin plus cyclophosphamide (group B1) or capecitabine (group B2) regimen and at completion of neoadjuvant chemotherapy. In group B, data of 2 patients (1 each in groups B1 and B2) who dropped out before cycle 4 are not shown. Asterisks (*) indicate patients with pathological complete response (CpCRypN0)

Figure 3 shows tumor size reduction in individual patients. In groups A1 and A2, 9 and 6 patients, respectively, had 100% tumor size reduction after initial paclitaxel- or eribulin-based regimen, and 13 and 10 patients, respectively, achieved CR after completion of anthracycline regimen (Fig. 3a). In groups B1 and B2, 2 patients and 4 patients, respectively, had 100% tumor size reduction after eribulin plus cyclophosphamide or capecitabine regimen (Fig. 3b).

Tumor size reduction from the start of anthracycline to completion of neoadjuvant chemotherapy is shown in Supplementary Fig. S2.

In group A, tumor regression in response to subsequent anthracycline in the patients who had initially received paclitaxel (group A1, *n* = 12) and those who had received eribulin (group A2, *n* = 14) was –56.0 (95% CI, –77.6 to –34.5) and –53.5 (–76.7 to –30.3), respectively. The mean change in tumor size at completion of neoadjuvant chemotherapy tended to be similar between groups A1 and A2 (Supplementary Fig. S3).

#### Breast-conserving surgery rate

In groups A1 and A2, 8 of the 23 patients and 13 of the 22 patients, respectively, underwent BCS. Point estimates were 26% (95% CI, 10%–48%) for group A1 and 55% (95% CI, 32%–76%) for group A2. In group B1 and B2, 9 of the 24 patients and 13 of the 25 patients, respectively, underwent BCS. Point estimates were 29% (95% CI, 13%–51%) for group B1 and 40% (95% CI, 21%–61%) for group B2.

#### Axillary lymph node status

The numbers of previously lymph node-positive patients who were lymph node-negative at surgery were 7 of 8 in group A1 and 6 of 8 in group A2, and 5 of 11 in group B1 and 2 of 10 in group B2 (Table 5).

**Table 5:** Proportion of patients whose axillary lymph node status had become negative by the time of surgery^a^

Group	N( +) at screening	Underwent surgery	ypN0	N( +) at screening and underwent surgery	N( +) at screening and underwent surgery, and achieved ypN0	Axillary lymph node status had become negative, % (95% CI^b^)	*P*^c^
A1 (*n* = 23)	8	23	19	8	7	88 (47–100)	1.00
A2 (*n* = 22)	8	22	16	8	6	75 (35–97)	
B1 (*n* = 27)	11	24	17	9	5	45 (17–77)	0.36
B2 (*n* = 27)	10	25	15	9	2	20 (3–56)	

^a^Values are *n* unless otherwise stated

^b^Clopper–Pearson exact confidence interval

^c^Fisher’s exact test: group A1 versus A2 or group B1 versus group B2

### Adverse events

The results for AEs are summarized in Table 6. In group A, the incidence of peripheral sensory neuropathy was lower in patients who received the eribulin-based regimen than in those who received the paclitaxel-based regimen (32% and 74%, respectively). All cases of serious AEs improved or resolved with appropriate treatment. No deaths were recorded. Overall, the incidence of AEs was similar to in previous studies.

**Table 6:** Incidence of adverse events^a,b^

Adverse event	Group A1 (*n* = 23)		Group A2 (*n* = 22)		Group B1 (*n* = 27)		Group B2 (*n* = 27)	
	Total	Grade ≥ 3	Total	Grade ≥ 3	Total	Grade ≥ 3	Total	Grade ≥ 3
All adverse events	23 (100)	19 (83)	22 (100)	20 (91)	27 (100)	24 (89)	27 (100)	25 (93)
Decreased neutrophil count	19 (83)	13 (57)	19 (86)	16 (73)	25 (93)	23 (85)	25 (93)	22 (81)
Decreased white blood cell count	17 (74)	5 (22)	15 (68)	10 (45)	24 (89)	15 (56)	21 (78)	11 (41)
Febrile neutropenia	4 (17)	4 (17)	7 (32)	7 (32)	3 (11)	2 (7)	2 (7)	2 (7)
Anemia	18 (78)	6 (26)	18 (82)	4 (18)	11 (41)	0	6 (22)	0
Thrombocytopenia	8 (35)	1 (4)	11 (50)	2 (9)	0	0	6 (22)	0
Alopecia	23 (100)	0	18 (82)	0	27 (100)	0	23 (85)	1 (4)
Constipation	19 (83)	0	20 (91)	0	16 (59)	0	16 (59)	0
Nausea	17 (74)	0	16 (73)	0	18 (67)	0	16 (59)	0
Increased ALT	10 (43)	0	16 (73)	3 (14)	14 (52)	6 (22)	16 (59)	4 (15)
Increased AST	10 (43)	0	16 (73)	0	11 (41)	0	17 (63)	3 (11)
Stomatitis	11 (48)	0	16 (73)	1 (5)	12 (44)	0	9 (33)	2 (7)
Dysgeusia	9 (39)	0	8 (36)	0	13 (48)	0	11 (41)	0
Malaise	9 (39)	0	10 (45)	0	10 (37)	0	10 (37)	0
Peripheral sensory neuropathy	17 (74)	0	7 (32)	0	6 (22)	0	7 (26)	0
Pyrexia	12 (52)	0	8 (36)	0	8 (30)	0	4 (15)	0
Decreased appetite	5 (22)	0	7 (32)	0	10 (37)	0	7 (26)	0
Diarrhea	8 (35)	0	3 (14)	0	6 (22)	0	8 (30)	0
Vomiting	8 (35)	0	10 (45)	0	4 (15)	0	2 (7)	0
Headache	5 (22)	0	7 (32)	0	3 (11)	0	7 (26)	0
Increased GGT	4 (17)	0	4 (18)	0	7 (26)	3 (11)	4 (15)	3 (11)
Insomnia	5 (22)	0	7 (32)	0	3 (11)	0	3 (11)	0
Peripheral and facial oedema	5 (22)	0	3 (14)	0	4 (15)	0	5 (19)	0
Fatigue	5 (22)	0	3 (14)	0	2 (7)	0	2 (7)	0
Hand-foot syndrome	0	0	0	0	0	0	11 (41)	1 (4)
Upper respiratory tract infection	6 (26)	0	2 (9)	0	1 (4)	0	2 (7)	0
Acne-like dermatitis	5 (22)	0	3 (14)	0	1 (4)	0	2 (7)	0
Arthralgia	4 (17)	0	5 (23)	0	1 (4)	0	0	0
Rash	3 (13)	0	5 (23)	0	0	0	1 (4)	0
Lymphocyte count decreased	1 (4)	1 (4)	3 (14)	3 (14)	3 (11)	0	0	0

ALT, alanine aminotransferase; AST, aspartate aminotransferase; GGT, γ-glutamyl transferase

^a^Adverse events of any grade occurring in ≥ 20% or grade ≥ 3 events recorded for ≥ 10% of patients

^b^Values are *n* (%)

## Discussion

In the present study, the number of enrolled patients did not reach the target sample size. This may be partly because other studies enrolling TNBC patients were ongoing at about the same time. Also, the study procedures, including HRD testing, were time-consuming (e.g. it took about 3 weeks to obtain the test results and determine HRD status by CPR), which made it difficult to obtain consent from patients who wished to start treatment immediately. However, the requisite number of patients for the analyses of safety and efficacy of the new regimens was eventually enrolled and data from a randomized phase II trial were obtained. To our knowledge, this is the first study that evaluated neoadjuvant eribulin for TNBC based on stratification by HRD score and gBRCAm status in the prospective randomized setting.

### Clinical usefulness of the eribulin–carboplatin neoadjuvant regimen

In group A, we investigated the standard paclitaxel or eribulin plus carboplatin regimens, each followed by FEC or AC. Recently, a study of neoadjuvant eribulin followed by FEC has been reported; pCR was achieved by a relatively low proportion of patients (8/43, 19%) [42]. Our study is new in that carboplatin was added to the initial paclitaxel- or eribulin-based therapy based on stratification of patients by HRD score and gBRCAm status; in both cases this resulted in high pCR rate (65% and 45%, respectively). Mean relative dose intensity for paclitaxel, eribulin, and carboplatin exceeded the recommended minimum of 80%.

A recent meta-analysis (nine randomized controlled trials, N = 2109) has shown pCR to be significantly higher in TNBC patients treated with platinum-based as opposed to platinum-free neoadjuvant chemotherapy (52.1% versus 37.0%) [43]. In the phase II study of neoadjuvant carboplatin plus eribulin for TNBC, HRD score and HRD status were found to be predictors of pCR in an exploratory analysis [32]. Our results support these findings, confirming the usefulness of HRD score to prospectively identify patients most likely to respond to the platinum-containing regimen. However, because of the small sample size and the exploratory nature of this study, care is necessary when interpreting the results. The ongoing translational study is expected to clarify the role of biomarkers.

We also found that, after the initial paclitaxel- or eribulin-based therapy, changes in tumor size at completion of neoadjuvant chemotherapy were similar between groups A1 and A2. The findings suggest the clinical usefulness of the new regimen in this population in terms of the pCR and tumor regression.

### Toxicity of eribulin- and paclitaxel-based regimens

Regarding safety, peripheral sensory neuropathy is a common AE of microtubule-targeting agents. In the present study, the eribulin-based regimen more than halved the incidence of peripheral sensory neuropathy with the standard paclitaxel-based regimen (32% and 74%, respectively). It affected less than a third of patients who received eribulin and was grade ≥ 3 in none. This is consistent with the findings of previous studies, in which peripheral neuropathy was recorded for about a third of pretreated breast cancer patients who received eribulin monotherapy and was grade ≥ 3 in < 10% [24, 44, 45]. We consider this lower-toxicity profile to be a benefit for TNBC patients for whom AEs are likely to have a greater negative impact.

The low peripheral neuropathy incidence was also observed in group B, in which eribulin was given in combination with cyclophosphamide or capecitabine (22% and 26% in groups B1 and B2, respectively). However, these regimens resulted in a low pCR rate (19% in each group), which was below the threshold pCR rate (35%). In a previous study, neoadjuvant eribulin plus cyclophosphamide resulted in a pCR rate of 13% in patients with invasive HER2-negative breast cancer [46]. Although our aim was to explore a less toxic regimen in this group, for which encouraging data were obtained, the finding of low pCR rate suggests these regimens to be unfeasible. Potential factors that may have affected sensitivity to chemotherapy in this group are low HRD score, tumors of lower histological grade (grade 3 in 67% and 44% in groups B1 and B2, respectively, versus 70% and 77% in groups A1 and A2, respectively), and older age (mean, ~ 60 years in group B versus ~ 46 years in group A). Because of the small sample size and phase II nature of the study, it is not possible to perform analyses to identify associations between these factors and the response. Further analyses, including long-term outcome data, may be needed.

The tolerability profiles of the eribulin-based regimens used in the present study were similar to those reported previously for eribulin-based regimens [24, 25] and FEC–docetaxel combinations [10–12], and there were no novel or unexpected AEs. All serious AEs were manageable, and there were no deaths related to treatment.

### Secondary endpoints

As a secondary endpoint, BCSR was almost doubled with the eribulin-based regimen as compared with the standard paclitaxel-based regimen [point estimates: 26% (95% CI, 10%–48%) and 55% (95% CI, 32%–76%) in groups A1 and A2, respectively]. Tumor reduction is generally associated with increased BCSR. However, it should be noted that the decision between mastectomy and BCS is affected by many factors, including tumor site and resection area, breast size, risk of gBRCAm, widespread use of post-surgical breast reconstruction, and patient preference. These factors make it increasingly difficult to use BCSR as a measure of the success of neoadjuvant chemotherapy in clinical trials.

A more objective measure than BCSR for evaluating neoadjuvant therapy is the proportion of patients whose lymph node status changes from positive to negative. Changes in lymph node status reflect the efficacy of a chemotherapeutic regimen and can greatly influence prognosis [47]. In the present study, the likelihood of negative lymph node status being achieved by the time of surgery was similar between the regimens in each of groups A and B. The long-term prognosis of the patients is being followed to explore the effect of changes in lymph node status on prognosis.

## Limitations

For the present study, fewer patients were enrolled than expected, and it took almost 2 years to recruit the requisite number. Nevertheless, we believe that our findings add to the literature suggesting potential benefits of clinically feasible and less toxic neoadjuvant chemotherapy for TNBC. A translational study is ongoing to investigate predictors of response.

## Conclusions

In patients aged < 65 years with high HRD score or gBRCAm, weekly paclitaxel plus carboplatin and eribulin plus carboplatin followed by anthracycline resulted in a pCR rate of > 60% and > 40%, respectively, suggesting the potential usefulness of patient stratification using HRD score. In patients with HRD-negative tumors, pCR tended to be low. Neurotoxicity was less frequent with the eribulin-based regimen, which may be feasible as neoadjuvant chemotherapy for TNBC patients for whom AEs are likely to have a greater negative impact.

## Supplementary Information

Below is the link to the electronic supplementary material.

Supplementary file1 (docx 22 kb)

Supplementary file2 (pptx 121 kb)
