# Supplementary material for: Eribulin-based neoadjuvant chemotherapy for triple-negative breast cancer patients stratified by homologous recombination deficiency status: a multicenter randomized phase II clinical trial
Source: Breast Cancer Res Treat. 2021 Mar 25;188(1):117–31. doi: 10.1007/s10549-021-06184-w (PMC8233289; doi:10.1007/s10549-021-06184-w)
Supplement: Supplementary file 1 — Supplementary file1 (docx 22 kb) [file 10549_2021_6184_MOESM1_ESM.docx]

# **Supplementary information**

**Inclusion and exclusion criteria**

**Inclusion criteria for primary registration**

Patients who fulfilled the following criteria were eligible for the primary registration:

1. Age 20–70 years at time of informed consent.
2. Primary breast cancer diagnosed as invasive breast cancer by a needle or vacuum-assisted breast biopsy (excluding patients with apocrine or medullary carcinoma).
3. Resectable primary breast cancer (cT1c-cT3, cN0-cN1, and cM0) with a tumor size ≤70 mm in diameter.
4. Triple-negative breast cancer [estrogen receptor (ER) <1%, progesterone receptor (PgR) <1%, and human epidermal growth factor receptor 2 (HER2)-negative] or weakly hormone receptor–positive breast cancer (ER <10%, PgR <10%, and HER2-negative) status confirmed in the primary lesion, and Ki67 labelling index ≥10% (if available).
5. No previous treatment for breast cancer.
6. Indicated for neoadjuvant chemotherapy.
7. Possible to evaluate the primary lesion using the same imaging test (i.e. contrast-enhanced MRI or PET/CT) throughout the period before, during and after the study treatment.
8. Possible to evaluate the primary lesion using mammary ultrasonography throughout the period before, during, and after the study treatment.
9. Written informed consent obtained from the patient.

**Inclusion criteria for secondary registration**

Patients who fulfilled the following criteria in addition to the primary registration criteria were eligible for the secondary registration:

1. Eastern Cooperative Oncology Group performance status 0**–**1.
2. The following conditions have been confirmed by the centralized pathologic review (CPR): invasive breast cancer; triple-negative breast cancer (ER <1%, PgR <1%, and HER2-negative) or weakly hormone receptor-positive breast cancer (ER <10%, PgR <10%, and HER2-negative) status; and Ki67 labelling index ≥10% (if available).
3. Homologous recombination deficiency (HRD) score (by Myriad Genetics, Salt Lake City, Utah, USA) for patients aged <65 years at time of informed consent (excluding patients with known germline *BRCA* mutation, gBRCAm).
4. Adequate organ function based on specified laboratory values.
5. In patients with HRD-negative tumor or those aged ≥65 years at time of informed consent (excluding patients with known gBRCAm), creatinine clearance ≥50 mL/min.
6. No clinically significant arrhythmia as determined by 12-lead ECG (QTc: ≤480 ms).
7. No interstitial pneumonia or lung fibrosis diagnosed by chest CT or radiography.
8. Non-pregnancy confirmed by a pregnancy test.
9. Patients with a possibility of pregnancy must agree to take contraception throughout the study period and until 2 months after the last dose.
10. HBs antigen, HBc antibody, and HBs antibody tests are all negative.

**Exclusion criteria**

Patients who met any of the following criteria were excluded from the study enrollment:

1. Diagnosis of bilateral invasive breast cancer.
2. Having multiple cancers other than breast cancer.
3. Having axillary lymph node dissection before start of neoadjuvant chemotherapy (sentinel lymph node biopsy was permitted).
4. Having incisional or excisional biopsy for the primary lesion or axillary lymph node (small incisional resection in patients with cT3 was permitted).
5. Participation in another clinical trial in the 28 days before the secondary registration.
6. Peripheral neuropathy of grade ≥2.
7. Cardiopulmonary dysfunctions in the 6 months before the secondary registration.
8. Myocardial infarction in the 12 months before the secondary registration
9. Severe uncontrolled systemic diseases (clinically significant cardiovascular, pulmonary, metabolic, or wound healing dysfunction or hypertension).
10. Underwent major surgery or had serious trauma in the 28 days before the secondary registration, or those with planned major surgery during the trial.
11. Severe infectious disease requiring intravenous administration of antibiotics, antiviral agents, or antifungal agents at the time of secondary registration.
12. Dental caries and/or oral infections requiring treatment.
13. Diagnosis of active liver disease or sclerosing cholangitis caused by autoimmune liver disorders.
14. HIV infection.
15. Lactating patients.
16. Other diseases affecting informed consent or protocol compliance determined by the investigator.
17. Hypersensitivity to the study drug or additives or dihydropyrimidine dehydrogenase deficiency.
18. Difficulty in oral administration of drugs including capecitabine, dysfunction of the upper gastrointestinal tract or malabsorption syndrome (only applies to patients with HRD-negative tumor or those aged ≥65 years at time of informed consent).
19. Other conditions determined to be ineligible by the investigator.
