# Supplementary material for: Eribulin-based neoadjuvant chemotherapy for triple-negative breast cancer patients stratified by homologous recombination deficiency status: a multicenter randomized phase II clinical trial
Source: Breast Cancer Res Treat. 2021 Mar 25;188(1):117–31. doi: 10.1007/s10549-021-06184-w (PMC8233289; doi:10.1007/s10549-021-06184-w)
Supplement: Supplementary file 2 — Supplementary file2 (pptx 121 kb) [file 10549_2021_6184_MOESM2_ESM.pptx]

## Slide 1
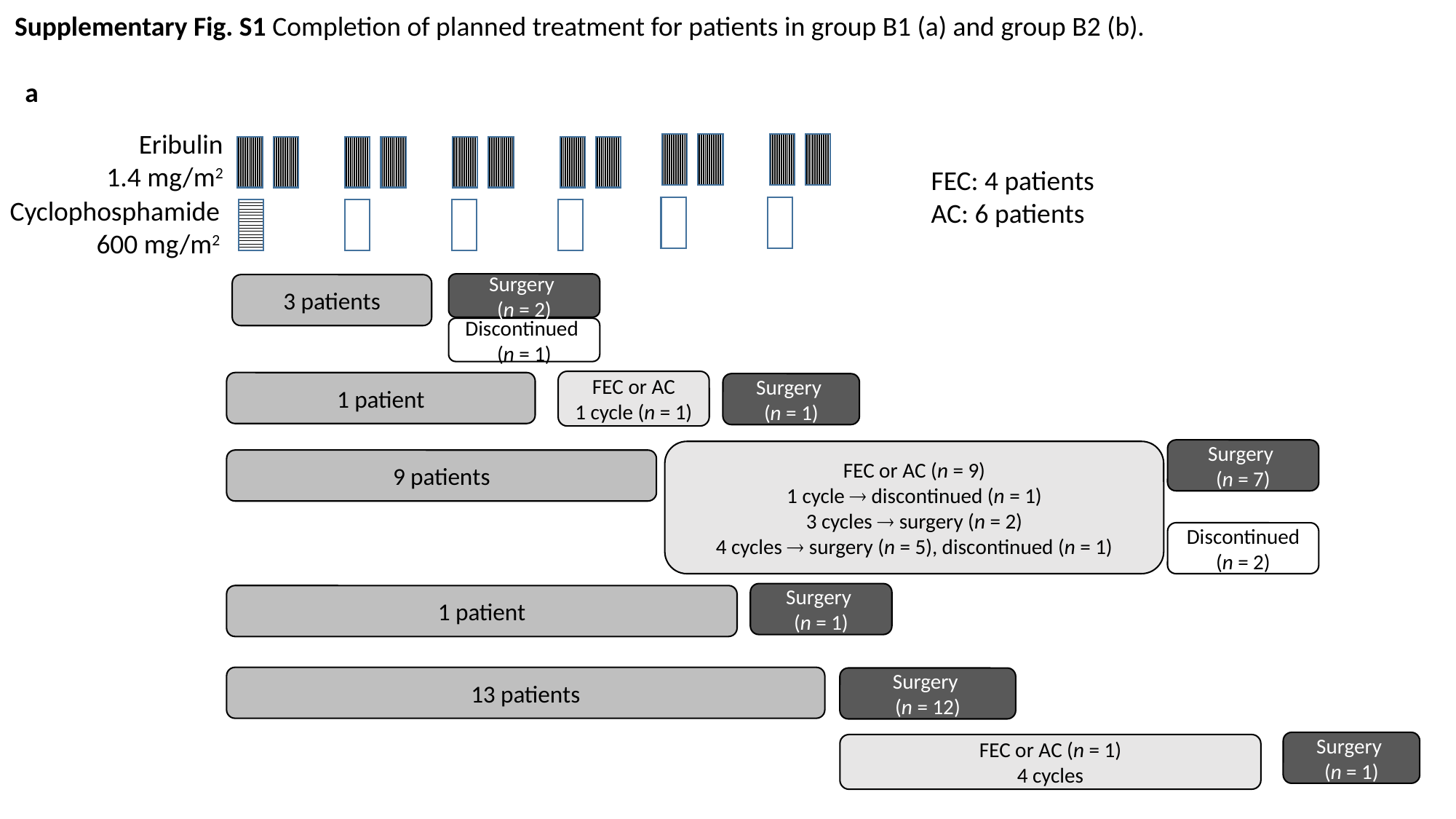

Supplementary Fig. S1 Completion of planned treatment for patients in group B1 (a) and group B2 (b).
a
Eribulin
1.4 mg/m2
FEC: 4 patients
AC: 6 patients
Cyclophosphamide
600 mg/m2
Surgery
(n = 2)
3 patients
Discontinued
(n = 1)
FEC or AC
1 cycle (n = 1)
1 patient
Surgery
(n = 1)
Surgery
(n = 7)
FEC or AC (n = 9)
1 cycle  discontinued (n = 1)
3 cycles  surgery (n = 2)
4 cycles  surgery (n = 5), discontinued (n = 1)
9 patients
Discontinued (n = 2)
Surgery
(n = 1)
1 patient
13 patients
Surgery
(n = 12)
Surgery
(n = 1)
FEC or AC (n = 1)
4 cycles

## Slide 2
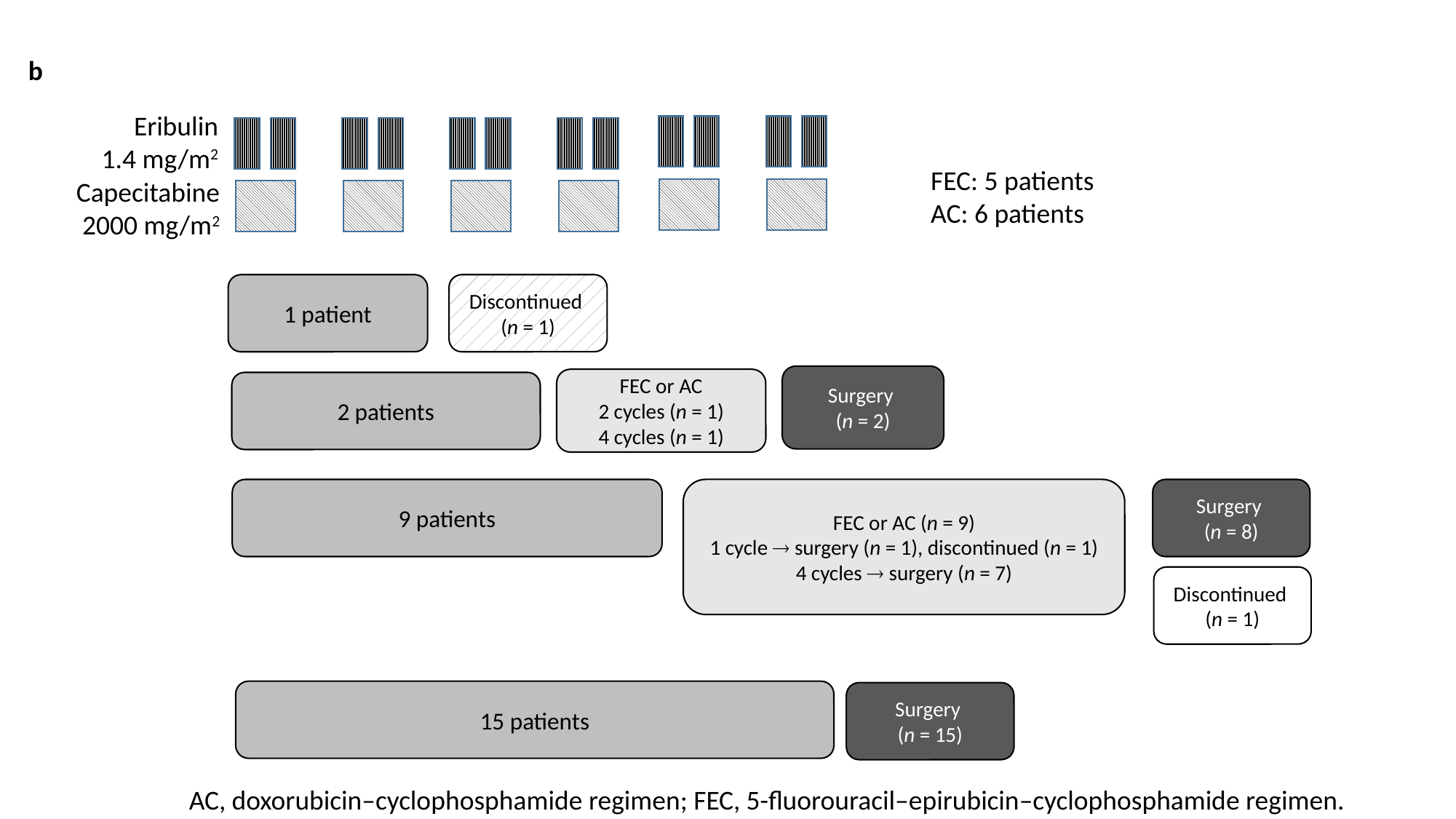

b
Eribulin
1.4 mg/m2
Capecitabine
2000 mg/m2
FEC: 5 patients
AC: 6 patients
1 patient
Discontinued
(n = 1)
Surgery
(n = 2)
FEC or AC
2 cycles (n = 1)
4 cycles (n = 1)
2 patients
Surgery
(n = 8)
9 patients
FEC or AC (n = 9)
1 cycle  surgery (n = 1), discontinued (n = 1)
4 cycles  surgery (n = 7)
Discontinued
(n = 1)
15 patients
Surgery
(n = 15)
AC, doxorubicin–cyclophosphamide regimen; FEC, 5-fluorouracil–epirubicin–cyclophosphamide regimen.

## Slide 3
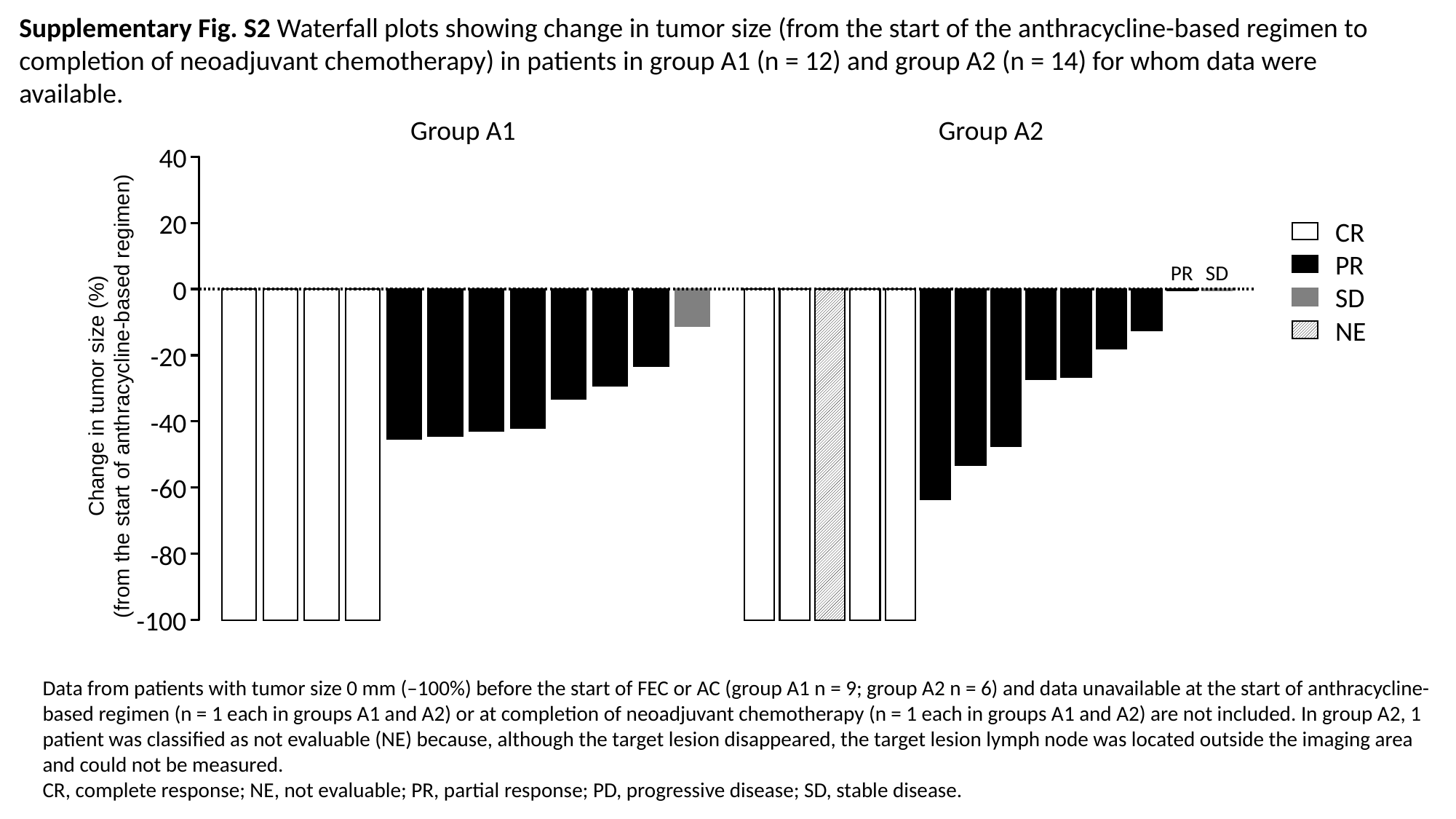

Supplementary Fig. S2 Waterfall plots showing change in tumor size (from the start of the anthracycline-based regimen to completion of neoadjuvant chemotherapy) in patients in group A1 (n = 12) and group A2 (n = 14) for whom data were available.
Group A1
Group A2
40
20
CR
PR
PR
SD
0
SD
NE
-20
Change in tumor size (%)
(from the start of anthracycline-based regimen)
-40
-60
-80
-100
Data from patients with tumor size 0 mm (–100%) before the start of FEC or AC (group A1 n = 9; group A2 n = 6) and data unavailable at the start of anthracycline-based regimen (n = 1 each in groups A1 and A2) or at completion of neoadjuvant chemotherapy (n = 1 each in groups A1 and A2) are not included. In group A2, 1 patient was classified as not evaluable (NE) because, although the target lesion disappeared, the target lesion lymph node was located outside the imaging area and could not be measured.
CR, complete response; NE, not evaluable; PR, partial response; PD, progressive disease; SD, stable disease.

## Slide 4
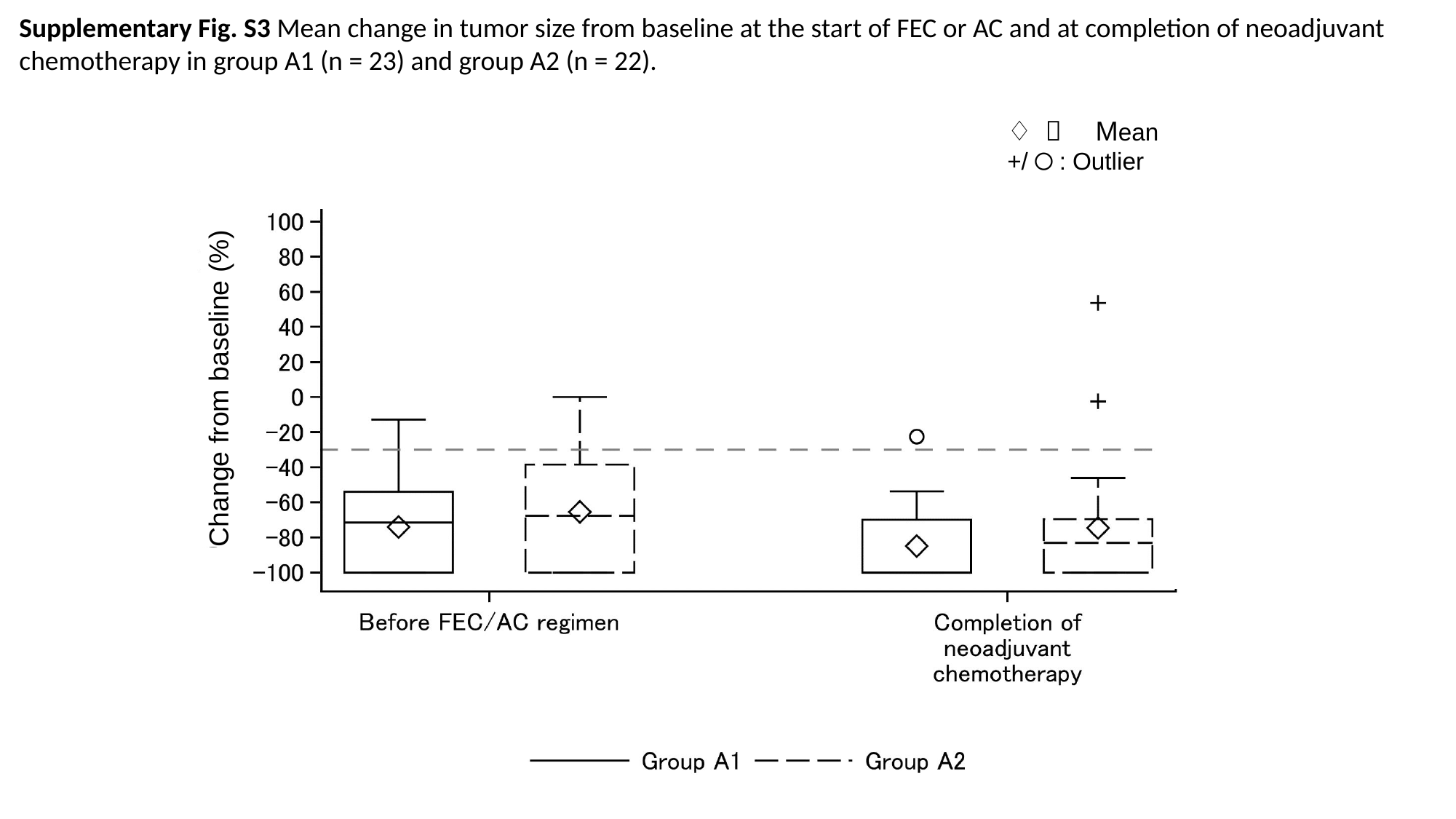

Supplementary Fig. S3 Mean change in tumor size from baseline at the start of FEC or AC and at completion of neoadjuvant chemotherapy in group A1 (n = 23) and group A2 (n = 22).
♢ ：　Mean
+/〇: Outlier
Change from baseline (%)
